# Influence of the Environment on the Reliability of Security Magnetic Contacts

**DOI:** 10.3390/mi12040401

**Published:** 2021-04-05

**Authors:** Martin Boros, Andrej Velas, Viktor Soltes, Jacek Dworzecki

**Affiliations:** 1 Department of Security Management, Faculty of Security Engineering, University of Zilina, Univerzitna 8215/1, 010 26 Zilina, Slovakia; andrej.velas@fbi.uniza.sk (A.V.); viktor.soltes@fbi.uniza.sk (V.S.); 2 Faculty of Security Science, Military University of the Land Forces in Wroclaw, Piotra Czajkowskiego 109, 51-147 Wrocław, Poland; jacekdworzecki@o2.pl

**Keywords:** magnetic contacts, reliability, practical tests, reaction distance, extreme conditions

## Abstract

Magnetic contacts are one of the basic components of an alarm system, providing access to buildings, especially windows and doors. From long-term reliability tests, it can be concluded that magnetic contacts show sufficient reliability. Due to global warming, we can measure high as well as low ambient temperatures in the vicinity of magnetic contacts, which can directly affect their reliability. As part of partial tests, research into the reliability of magnetic contacts, we created a test device with which their reaction distance was examined under extreme conditions simulated in a thermal chamber. The results of the practical tests have yielded surprising results.

## 1. Introduction

Magnetic contacts and magnetic fields have become an integral part of our lives, even though most of the time we would not even realize it. Thanks to our advantageous capabilities, we have been using them for almost a hundred years. They were developed in 1936 in the laboratories of Bell Telephone Laboratories by Walter B. Elwood, and three years later, in 1940, the first patent record was recorded, which is almost identical to the magnetic contacts used today [1]. With the development of time, the possibilities of using magnetic contacts in various branches of industry gradually developed, from compasses to handling equipment, computer memories, hard disks, storages, payment cards, and many other possibilities. In addition to magnetic contacts, formed by a permanent magnet and a reed contact, magnetic loops are also very often used in practice. Within the scientific community, the magnet or its components have been used by several scientists, such as Macholí Belenguer and his team [2], which have explored the possibilities of identifying vehicles by changing the voltage in the magnetic loops. On the other hand, the authors Primin and Nedayvoda [3] focused their research on the possibilities of using magnetic fields in biological identification using a SQUID sensor, while Ponce et al. [4] dealt with the efficiency of using magnetic sensors in water meters. However, these and other important scientific works suggest that the importance of magnetic contacts in science is very important.

The security sector is no exception, in which several possibilities of magnetic contact use are available, such as in the case of control of inputs in which magnetic cards are used or as detectors in an electrical security system [5,6]. It is the latter option that is one of the basic options for securing entrance openings within the building envelope, such as doors or windows. Thanks to their characteristics and the possibility of use, they are also used in perimeter protection (securing the entrance/exit gate from the premises) or also object protection. As mentioned, the base of the magnetic contacts is formed by a permanent magnet and a tongue, the ferromagnetic contacts of which are encased in a thin glass tube with a protective atmosphere to prevent corrosion. The magnetic part is usually installed on a moving part such as a window, door, or other objects whose change of state triggers an alarm. The tongue or contact is mounted on a fixed part, usually a door frame. From a functional point of view, we then divide the magnetic contacts into normally open (NO) and normally closed (NC) [1,6,7].

**Figure 1:**
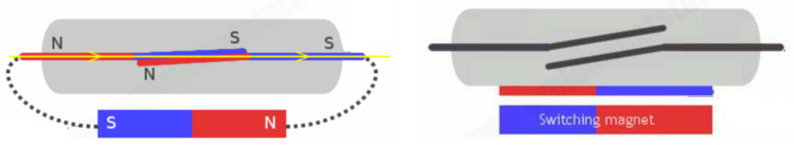
Example of making magnetic contacts on the left type of normally open (NO), on the right type of normally closed (NC).

The NO magnetic contacts close when the switching magnet approaches, allowing the passage of electrical current. On the contrary, the NC contacts are closed without the action of a magnetic field and allow the passage of an electric current. This is because they contain another magnet. The closing of the magnets is caused by the addition of another magnet, i.e., another permanent magnet is added to the NO type [1,8]. The difference in the evaluation of the magnetic contacts is shown in Figure 1.

The actual spacing of the contact parts is only a few microns, so these contacts are very sensitive. The contacts are manufactured in different versions for different security levels and environmental classes. We know magnetic contacts in metal and plastic housings as well as contacts intended for surface mounting or drilling mounting. A special group is the magnetic contacts connected to the control panel wirelessly. Magnetic contacts can be overcome in several ways, especially by using a parasitic magnet with a high magnetic field strength or by bridging the cabling. For these cases, sabotage circuits consisting of differently oriented magnetic contacts are also inserted into magnetic contacts intended for higher security classes [1,8,9].

As mentioned above, magnetic contacts represent one of the basic types of alarm system detectors, and therefore all the same requirements apply to them as to other elements of the system. It is usually a matter of defining the degree of security and the environment class, according to the European technical standard EN 50131-1 and the separate functional requirements comprehensively stated in the European technical standard EN 50131-2-6 determined by magnetic contact [9,10,11]. Security levels, according to EN 50131-1, are divided into four categories with a designation from 1 (lowest risk) to 4 (high risk). The four types are also divided into categories, environmental classes, the first two of which are intended for indoor and the second two for external environments [10]. The user should follow the manufacturer’s recommendations and use the detectors only in the recommended environment class and at the required security level. Failure to correctly classify the environment may result in a malfunction of the detector, and thus of the electrical system as a whole [6]. According to [11], the reliability parameters of the magnetic contact must be met to a tolerance of +/−10%, unless exceptions are defined. The mentioned standard defines several possibilities of performing functional tests of magnetic contacts to determine the correct functionality from the point of view of electrical requirements, switching requirements, detection requirements. A separate group of tests of magnetic contacts—as well as other detectors of intruder alarm systems—consists of environmental tests, which are addressed in the European technical standard EN 50130-5 [11,12].

The need for reliability testing in extreme ambient conditions is significant mainly due to global warming, in which we have witnessed extreme fluctuations in temperature values in the mid-range in recent years. In recent years, the number of research focusing on extreme weather conditions has also increased, as people realize the need to test different types of systems under ambient loads. For example, in the conditions of the Slovak Republic, the deviation of average temperatures recorded in 2018 increased by 1.3 °C compared to previous years, which corresponds to the long-term average warming of Europe and the whole planet [13]. However, due to global pollination, we also encounter the so-called “Arctic winters”, when the temperature drops well below freezing. In the coldest village in Slovakia, Oravská Lesná, the lowest temperature in the last decade was measured in 2017 at −35.5 °C. In the long run, it is, therefore, appropriate to consider whether the definition of four environmental classes with a specific temperature range is sufficient, as in the highest class, class IV, a temperature range from −25 °C to +60 °C is calculated [10,12,13].

To investigate the influence of temperature on the magnetic characteristics of NdFeB permanent magnets, the authors Calin and Helerea investigated the influence of temperature on the demagnetization curves of cylindrical magnets in the temperature range from 25 °C to 120 °C. According to their findings, the properties of cylindrical magnets change at approximately 50 °C [14]. Our research aims to express the working distance of magnetic contacts within which it can detect the intruder even in degraded ambient conditions. We believe that despite the results of the mentioned research, the working distance would not have to be so affected. Within our assumptions, we are working with the knowledge that the magnetic properties are influenced not only by temperature but also by other influencing parameters such as the shape and material of the magnet or the surrounding metal objects. We also base our knowledge on the average, maximum operating temperature of magnetic contacts being 80 °C.

Due to extreme weather conditions, the design of a resilient distribution network was addressed by Shahbazi A and colleagues, who achieved very good system resilience results in their case studies [15]. Another interesting and significant research was conducted by Bennett, JA, and colleagues, who focused on modeling the optimization of energy system architecture as prevention against hurricane impacts [16]. We decided to research the influence of extreme weather influences on the reliability of magnetic contacts as one of the partial parts of comprehensive research into the components of electrical security systems. We did not rely on the conditions and recommendations of European technical standards, as we wanted to simulate real conditions where the perpetrator may not follow the procedures prescribed by the standard. We followed up on the research activities of the workplace, within which we focused on the evaluation of camera systems against extreme weather conditions, as well as many other important tests of transmission systems, communication interface, location system, and the like [16,17,18,19,20].

Experimental testing of extreme weather effects on camera systems has shown the fact that they can fully operate outside the temperature ranges specified in the technical standard. It was found that a camera system designed for the second class of environment, −10 °C to +40 °C, is able to fully operate at a temperature of +120 °C [13]. The problem, in this case, occurred at a value of +160 °C, when the system stopped and appeared to be without a camera connected. However, after sufficient cooling, the camera continued to function but showed permanent image damage due to sensor damage [21]. Based on the findings, we also decided to further verify the functionality of the security system components. Comprehensive and extensive findings could be used as a basis for adjusting the environmental classes specified in the technical standard for security systems. If further component testing is possible, the magnetic contacts should be used, as this is the most commonly used component of the security system, designed to protect the opening parts in the building or the premises of the building. Another reason for choosing magnetic contacts was their financial simplicity and the fact that they are the most important element, especially in the case of entering the building, which must function properly for the correct detection of the intruder. From the tests of camera systems, it is possible to use and focus only on the realistically achievable thermal values, while the limit temperature of the functionality of the magnet should not be exceeded. In the case of a camera system, such values were not and are not generally known, and therefore we went to huge extremes, but at the cost of long-term damage to the camera [10,13,21].

## 2. Materials and Methods

As part of the evaluation of the impact of extreme conditions, we decided to perform experimental measurements using our test equipment in the air conditioning chamber. Using experimental measurements, we investigated the causality between the effect of temperature and the closing/opening distances of five selected types of magnetic contacts intended for outdoor use.

The basic pillar of measurements was the mentioned air-conditioning chamber, brand VÖTSCH VCL 7010, which we have available within the material and technical equipment of the laboratory. The air conditioning chamber provides a temperature range from −70 °C to +180 °C and a heating and cooling rate of 3.5 K/min [22].

**Figure 2:**
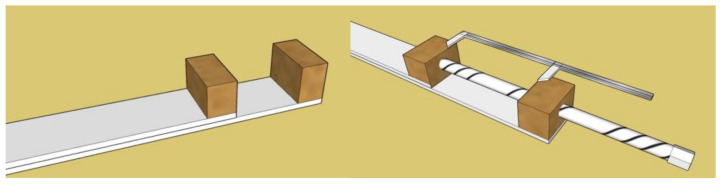
Graphic design of the test device from the top right, the drive part from the left.

We created a test device especially for this type of measurement, which consisted of two hardboard plates with a length of 50 cm and a width of 3 cm. At the ends of both boards, we attached two wooden prisms with glue, which were used to hold the tested magnetic contacts, and we placed the boards on top of each other. The model of the upper part of the test device was created in the Sketch UP! program, as shown in Figure 2. We decided to use wooden prisms, as they are a non-conductive material, and so will not affect the detection and switching functionality of the tested magnetic contacts. We also used glue rather than iron screws to attach them. For better handling of the test equipment, we connected the boards in several places with mounting tapes to perform only one direction of movement [23].

**Figure 3:**
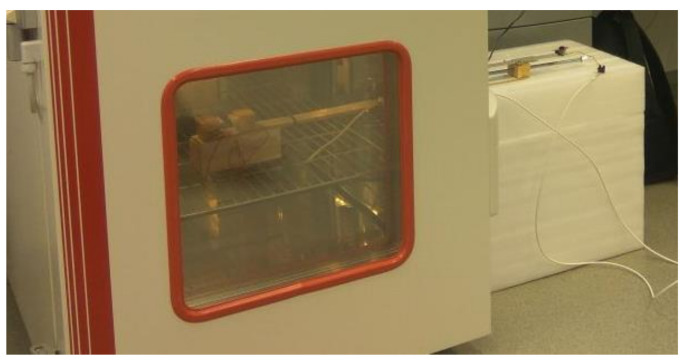
Test equipment placed in an air-conditioning chamber during experimental testing.

At the other end of the boards, we placed a drive, which was formed by a threaded rod with a diameter of 8 mm. With the help of one turn of the threaded rod, it was possible to move the wooden prisms by 1.25 mm, which achieved a very precise and sensitive mechanical movement. We achieved the drive-by drilling wooden prisms and then inserting a threaded screw into a hole on a fixed (bottom) plate. To achieve, in addition to movement, the measurement of the closing distance of the tested magnetic contacts, we also attached a caliper to the wooden prisms, with the help of which we were able to express the given distance. The model of the lower part of the test device together with the drive, created in the program Sketch UP! is shown in Figure 2. The test device in the air conditioning chamber is shown in Figure 3.

**Figure 4:**
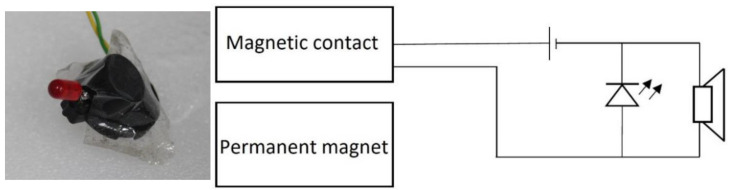
Indication device (right) and its wiring diagram.

We read the values manually from the caliper, so it was possible to assume that a measurement error had occurred. We rounded the values to 0.5 mm, which could cause a measurement error of ±0.25 mm. We designed the test device to be able to zoom in and out on the magnetic contacts in the air conditioning chamber. We adapted its size to the size of the chamber opening. We used an optical and acoustic indicator to determine the activity/inactivity of the magnetic contact. The indicator consisted of LEDs, piezoelectric buzzer with a power supply. We attached the indicator constructed in this way to the magnetic contact, and in the case of its switching on, i.e., activation, the LED lit up and the buzzer sounded. The figure of the indicator, as well as its wiring diagram, are shown in Figure 4.

As mentioned in total, we tested five different magnetic contacts in experimental tests and followed the following procedure for each measurement:

- Study of the enclosed technical sheet of the magnetic contact from the manufacturer,
- Fixing the magnetic contact to the test equipment, usually by double-sided adhesive tape or by impregnation into holes in wooden prisms,
- Implementation of experimental tests:
  ○ measuring the closing distance—movement from 0 mm to 100 mm,
  ○ measuring the opening distance—movement from 100 mm to 0 mm,
- Evaluation of the measurement process and achieved results.

We measured the temperature range from −40 °C to +70 °C, while at negative values we changed the temperature by 5 °C and in the case of positive values by 10 °C. For each heat value, we performed two types of measurements, switching on and off, according to the above procedure. We stopped for two minutes between the individual distance measurements to prevent the measurement from being devalued due to the lasting closing/opening action from the previous measurement. We proceeded in descending order from positive to negative values to avoid possible corrosion. We recorded the measured values and expressed the average values using the average function in MS Excel. Subsequently, we used the STDEV function in MS Excel to express the standard deviation. In the case of each temperature change, we allowed the magnetic contact to acclimatize for 10 min, and then we performed 10 repetitions of the magnetic contact closing measurement. The transition between temperatures was relatively fast, falling for tens of minutes. The values given in the results section represent the arithmetic mean of the measured values for the individual temperatures. For those magnetic contacts for which the closing and opening did not occur at the same distance, we also determined the hysteresis distance by the difference between the closing and opening distance.

## 3. Results

**Figure 5:**
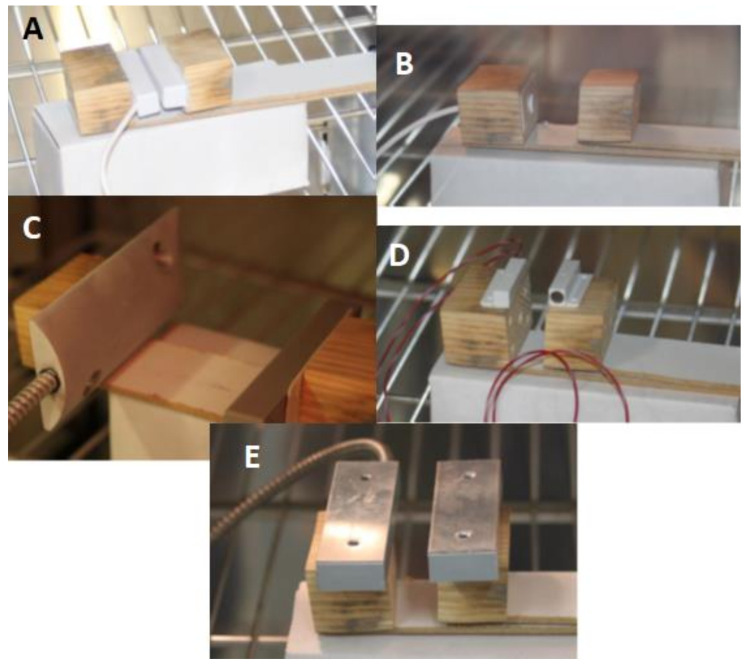
Magnetic contacts during experimental testing, (**A**)-MAS 203, (**B**)-MAS 333, (**C**)-SA 220, (**D**)-USP 131, (**E**)-USP 500.

We were the first to test the magnetic contact with the marked MAS 203 from the Czech manufacturer Asita. In this case, the manufacturer does not state the values of closing and opening of the magnetic contact, but only the working distance of 0–30 mm with a tolerance of 2 mm within which it should work fully. The installation requirements also state that the parallelism of the installation of the magnetic contacts must be observed. To ensure correct installation, we used double-sided adhesive tape, the anchoring of the magnetic contact during the measurement on the test device is shown in Figure 5A. The results obtained in the measurement are shown in Table 1.

As we can see due to the higher temperature, in particular, the working distance of the magnetic contact increased by 2–3 mm compared to the values given by the manufacturer, even with the permissible tolerance. Even for temperatures of 0 °C or temperatures around 20 °C (standard outdoor temperature), the distance was covered even when considering tolerance. On the contrary, negative values slightly reduced the working distance values. According to experimental tests of the reliability of a given magnetic contact, the average working distance was at the level of 30.5 mm [24]. By measuring, we found that the magnetic contact does not work with any opening hysteresis, i.e., the closing and opening of the magnetic contact occurs at the same distance [25]. Throughout the measurement, we followed the procedure created and determined by us, listed in Section 2.

The second magnetic contact tested was from the same manufacturer but with the type designation MAS 333. According to the information provided by the manufacturer, this type of magnetic contact can be used in environment-class III, i.e., in the temperature range from −25 °C to +50 °C. Since we followed the same measurement procedure for all magnetic contacts, we performed measurements at temperatures from −40 °C to +70 °C and these results are shown in Table 2. The working distance of the magnetic contact MAS 333 is 0–22 mm with tolerance according to the manufacturer 2 mm. As this is a countersunk type of magnetic contact, we used holes in the wooden prisms of the test device to attach it [26]. The location of the magnetic contact in the test device during the measurement is shown in Figure 5B.

**Figure 6:**
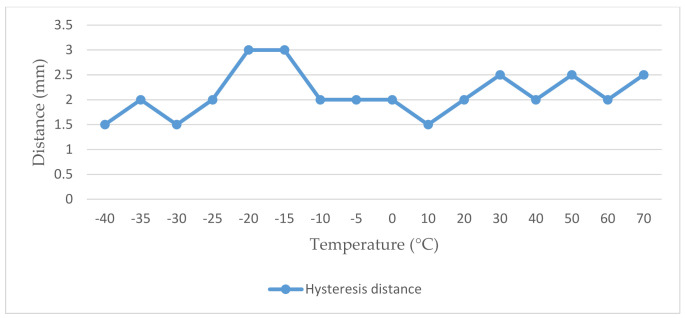
Graphical representation of the values of the hysteresis phenomenon of the magnetic contact MAS333.

From the achieved results we can state that the magnetic contact in case of closing always worked in the range of the working distance specified by the manufacturer, taking into account also the allowed tolerance. Conversely, in the case of opening the activation of the magnetic contact, we measured different values, exceeding the working distance, in the temperature range from −25 °C to +70 °C. The different values were caused by a hysteresis phenomenon, the values of which are shown in Figure 6 [23].

Another tested magnetic contact had the type of designation SA220 and is primarily offered by Jablotron in its portfolio. According to the information from the manufacturer, the magnetic contact is intended for environment-class IV, i.e., the highest class, with a temperature range from −25 °C to +60 °C. The body of the magnetic contact, the switching part, is housed in a metal housing and the permanent magnet in a plastic housing. Dimensionally, this is the largest magnetic contact with atypical dimensions and omnidirectional radiation, i.e., according to which axis within the three-dimensional space is installed, the distance of switching on and off is determined. As part of the measurements, we decided to use an installation with a *z*-axis, as due to its size, the manufacturer states the largest values of clamping (64 mm) and expansion (77 mm). We used double-sided adhesive tape for installation on the test equipment, the magnetic contact during the measurement is shown in Figure 5C. Even in this case, we worked with a temperature range from −40 °C to +70 °C.

**Figure 7:**
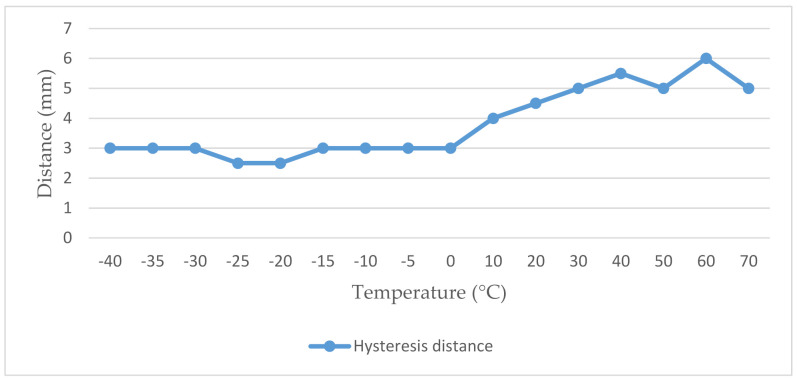
Graphical representation of the values of the hysteresis phenomenon of the magnetic contact SA 220.

As we can see in Table 3, we observe an almost exponential dependence of distance on temperature. While at negative values, the closing and opening distances were several tens of millimeters below the level specified by the manufacturer. Conversely, in the case of high temperatures, the values of switching on and off were much higher. Compliance with the data given by the manufacturer was achieved only in the temperature range −5 °C to 0 °C when closing and +10 °C to 20 °C when opening the magnetic contact. For measurement, we also recorded a significantly high value of hysteresis distance, in some cases at the level of up to 6 mm. The complete expression of the hysteresis distance is shown in Figure 7 [23,24,26]. The measurement results are quite worrying, as the magnetic contact did not work properly in the temperature range in which it was recommended to be installed.

Another magnetic contact tested came from United Security Products and was type-named USP 131. It is a small magnetic contact, almost 35 mm, housed in a plastic case. According to the manufacturer, the magnetic contact can work with a working distance of 20 mm, while it is designed for environment-class III, i.e., within the temperature range from −25 °C to +50 °C. Due to the uniformity and adherence to the established methodological procedure, we subjected the magnetic contact to reliability testing in the temperature range of −40 °C to +70 °C. The results obtained in the measurements are given in Table 4. The magnetic contact, USP 131, has an elongated part on the lower part of both components intended for their assembly, so we decided to place it on top of the test device. The location of the magnetic contact during the measurement is shown in Figure 5D.

**Figure 8:**
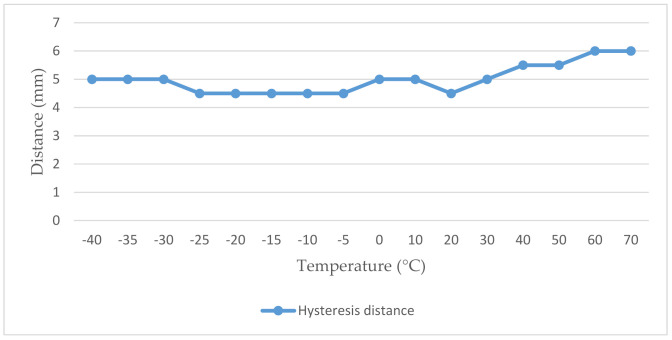
Graphical representation of the values of the hysteresis phenomenon of the magnetic contact USP 131.

As we can see from the achieved results, the manufacturer’s stated working distance of 20 mm was not reached in any of the measured temperatures. A positive finding is that the closing and opening distance of the magnetic contact was almost constant. Another positive is that the magnitude of the hysteresis distance that we observed when switching the magnetic contact is also, almost at a constant level, gradually increasing from a temperature of 30 °C [9,10]. The hysteresis distance is shown in Figure 8.

The last magnetic contact tested had the designation USP 500 and was therefore from the same American company as the previous contact. It is a relatively durable-looking magnetic contact designed for surface installation as it is formed by two iron, elongated blocks. In this case, too, it was a magnetic contact intended for environment-class III and was subjected to measurements at the same temperature levels as all other magnetic contacts. The working distance specified by the manufacturer is 63.5 mm, the attachment of the magnetic contacts during the measurement was realized with the help of double-sided tape and is shown in Figure 5E.

**Figure 9:**
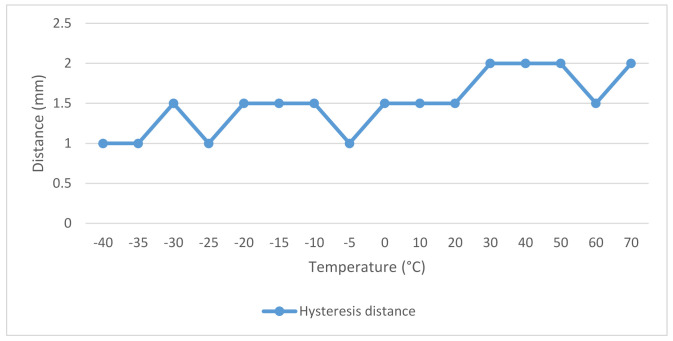
Graphical representation of the values of the hysteresis phenomenon of the magnetic contact USP 500.

As we can see from the results in Table 5, the magnetic contact did not even approach the working distance given by the manufacturer at sub-zero temperatures, at −40 °C, there was even a difference of almost 20 mm. An indication of the approach to the data from the manufacturer can be observed only at a temperature of 40 °C, but even in this case, the difference between the distances was at the level of 3 mm. When the switching element is switched on and off by a permanent magnet, it was possible to observe a hysteresis phenomenon of small size when compared to some of the other magnetic contacts. The values of the hysteresis phenomenon are shown in the graphic design in Figure 9.

## 4. Discussion

Through experimental tests that we performed in the air conditioning chamber, we can conclude that several of our assumptions have been confirmed. The reliability of the safety magnetic contacts is influenced by the shape of the magnetic contacts. We base this statement on the results in which we found that in the case of the four tested magnetic contacts, we measured relatively reliable data, within which the magnetic contacts would be able to function as expected. In one case, SA220, there was an appropriate difference as the measured values were almost 20 mm different than stated by the manufacturer. The possibility that the manufacturer incorrectly determined in the technical file the position of the sides of the magnet within the *x*, *y*, *z* axes would also be considered. However, even if we consider that we connected the magnetic contact oppositely, in both other possibilities the magnetic contact showed a difference of at least 15 mm in size. Based on these results, we must conclude that the magnetic contact is unreliable, even in the environment specified by the manufacturer.

In other cases of measurement, we achieved interesting results, in which we achieved almost identical results in all tested temperatures. By default, we achieved a difference of approximately 2 mm between negative and positive temperatures. It was confirmed that the cold benefits the functionality of the magnetic contacts, as we measured values similar to those stated by the manufacturer at negative values. Based on these results, we can conclude that the other four magnetic contacts are reliable despite the lower, measured distances. Most of the measured values were within the working distance specified by the manufacturer or within the 10% tolerance allowed by the technical standard [11]. We achieved interesting and surprising results in positive and high temperatures, respectively, where there was no rapid change in working distance as expected. It can be stated that we correctly assumed that within the standard operating temperature of the magnetic contacts, the shape of the block, the magnetic induction does not change to such an extent as to affect the reliability of the safety magnetic contacts.

**Figure 10:**
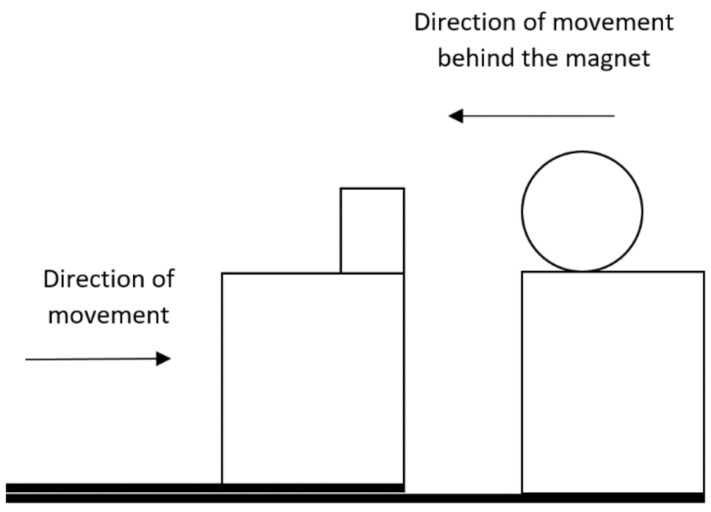
Block diagram of magnetic field strength measurement.

As they are undoubtedly interesting and new findings, we decided to perform a simple measurement to determine the strength of the permanent magnet, which is used to switch the magnetic contact. For the experiment, we used a test device created by us and a steel cylinder made of 18 washers, a screw, and a butterfly nut. The measurement consisted of setting the initial position, which was seven centimeters. We placed a permanent magnet on the sliding part of the test device and the mentioned steel cylinder on the fixed part, always so that the edge of the cylinder is aligned with the edge of the wooden prism. Gradually, we moved the test device until the moment when the steel cylinder, due to the attractive force of the magnet, did not start moving towards the permanent magnet. The block diagram of the measurement is shown in Figure 10. We performed the measurement at several temperatures and with twenty repetitions, the results of the measurement are shown in Table 6.

As can be seen from the measured values, the attractive force of the magnetic field does not change under the influence of temperature—or rather, changes to a negligible extent. For this simple measurement, we used a magnetic contact USP 131. The reduced force values, compared to Table 4, are caused by the weight of the steel cylinder, which was 200 g, and it was, therefore, necessary to approach it close enough to start moving. Due to the fact that it was a movement, it was necessary to carry out the measurement very carefully and precisely, which is also indicated by the value of the standard deviation.

Based on the findings from the results of the experimental tests given in Section 3 and the measurements described above, we can state that extreme temperatures in the standard working range of magnets do not affect the values of their intensity and field size. They only affect the switching capacity of the magnetic contacts. This effect is undoubtedly caused by the heating of a part of the magnetic contact such as screws for connecting cables or other components. These expand due to the high temperature, and therefore the connection/activation of the safety magnetic contacts takes a longer time or a greater distance. If we consider even more modern safety magnetic contacts in which more electronics are used, the distance could be even greater, because the temperature could affect the responsiveness of the safety magnetic contact, which would not be able to actively respond to closing or opening.

In the study, we also relied to a large extent on the results of the study by Calin and Helerea [14], but we were not able to confirm or refute their results, because our studies were significantly different. In our case, we focused on the switching ability of safety magnetic contacts, formed by two parts. On the contrary, the authors of the mentioned study measured magnetic quantities for one separate piece of the permanent magnet.

At first glance, it might seem that the magnetic contact, the USP 500, was also unreliable as it worked with a working distance almost 15 mm shorter. However, the opposite is true, as the magnetic contact worked correctly and within the operating range specified by the manufacturer. We have encountered a lack of working distances, namely that relatively wide ranges are mentioned and therefore the magnetic contact is considered reliable as long as it can be activated/switched on anywhere within the working distance.

The need to test the components of alarm systems is very important, as regular testing and evaluation of their reliability can predict the likelihood of their proper functioning [27,28]. In addition to the functional point of view, it is necessary to take into account the economic aspect, the value of which can be directly validated according to the needs of the secured object. Components of alarm systems are electrical devices for which the more expensive are not necessarily higher quality [29,30]. Knowledge of the probability of failure of a selected system component is very important for the effective design of the system. For example, if we know from the experimental tests the weaknesses of a selected component, we can increase its resistance by adding another component working on a different principle, thus achieving the component guarding the component. By partially increasing the security of a selected part of the building, we can increase the security of the entire building, according to its needs. The needs for object protection are an important input element in the evaluation and design of its security.

Knowing the reliability of individual system components is important because it allows us to choose from the same components on the market. All components should be certified and therefore usable in the conditions of the Slovak Republic. However, the certification takes place according to a predetermined methodological procedure, considers selected options for overcoming, and, last but not least, is performed under laboratory conditions. However, the potential perpetrator will not follow the methodological procedures or the technical standard permitted tools or objects when trying to overcome the security of the object [31,32]. Testing of alarm system components and detectors must be carried out as much as possible under non-standard conditions because only then is it possible to identify a higher degree of their reliability. Within the given partial part of the research of magnetic contacts, we focused on the simulation of extreme ambient conditions, as we encountered incorrect functionality of components several times. System malfunctions caused by extreme conditions can result in two basic possibilities. The first is the occurrence of false alarms and the second is the incorrect functioning of the system and thus the absence of guarding. In the first case, it is highly likely that the owner will be dissatisfied with the system due to multiple false alarms and could even partially deactivate the system, bringing us to the second option [33]. It is possible to test whether magnetic contacts or other components of alarm systems function according to the requirements of technical standards, but this would probably be unnecessary and we would get confirmation that they meet the requirements of the standard, as most components are certified.

## 5. Conclusions

Magnetic contacts are among the basic and most commonly used components of alarm systems. Their correct functionality is an integral part of the mantle protection of each secured object. As with other alarm system components, magnetic contacts are considered electrical devices and as such may not always achieve the required reliability. Reliability is often affected by restrictive conditions such as ambient temperature, poor installation, improper selection, and the like. As part of a comprehensive study of the reliability of magnetic contacts, we have decided to focus on the impact on the environment, as in recent years we have witnessed large fluctuations caused by global warming. Our goal was not to try to overcome safety magnetic contacts but to know their reliability at extreme temperatures, so we decided to perform only these types of measurements and avoided the use of parasitic magnets or other short-circuit options that can be used to overcome them.

We tested five magnetic contacts designed for external environments, environment classes III and IV, i.e., operating temperatures from −25 °C to +50/60 °C. In the tests themselves, however, we considered temperatures outside the temperature range. The temperature range we chose was from −40 °C to +70 °C within which we tested all components to achieve the same conditions. Through testing, we have found that temperature ranges in some cases cause a problem and, for example, in the case of magnetic contact USP 500, a shift of switching on at negative temperatures of 20 mm. In four of the five tested magnetic contacts, we observed a hysteresis phenomenon, which we could sometimes call directly proportional to the influence of the environment. We have found that magnetic contacts can work even in extreme conditions, but these to some extent affect their detection ability, which can decrease due to very low temperatures and increase at extremely high temperatures. Thus, in addition to the hysteresis, we can also observe the expandability of the material. Paradoxically, the magnetic contacts placed in the plastic housing showed a lower degree of hysteresis than the metal ones. To be able to carry out such tests, we created a simple test device, which, in addition to protecting the lives of researchers, also eliminated possible errors caused by heat radiation from the human body.

The magnetic contact behaved best in the tests with the type designation MAS 333, which during the change of temperatures showed an almost constant closing distance and the value of the hysteresis phenomenon was in the range of a maximum of 2 mm. On the contrary, the worst hit was the magnetic contact with the type designation SA 220, which in the case of switching on showed a difference of almost 30 mm and opening of almost 20 mm.

Through experimental tests, we probably came to the direct influence of the magnetic contact design on its reliability, as all square-shaped magnetic contacts showed good properties and activation within the working distances defined by the manufacturer or within a tolerance of 10%. Even the statistical deviation in these measurement cases was at a relatively low level, which can be considered as an effective measurement result. We recorded surprising results in the case of positive, high temperatures within which the magnetic contacts continued to work correctly and there was no rapid change in reliability or detection distance. Undoubtedly significant was the fact that we performed measurements in the temperature range up to 80 °C, i.e., the temperature considered critical. After this temperature, there could be a permanent change in the functionality of the magnetic contact or the permanent magnet. In the future, we would like to focus on this type of experimental test. The behavior of the hardened plastic into which the individual parts of the safety magnetic contacts are caught is also questionable.

It would also be possible to build on the results of our research and supplement the research with a comprehensive measurement of magnetic induction at high temperatures. This would confirm or refute the conclusions about the expansion of the materials used in the switching part of the magnetic contacts. The advantage, and at the same time disadvantage, is the fact that there are countless safety magnetic contacts on the market and, to fulfill our long-term goal, to amend the technical regulations. It will therefore be necessary to carry out a huge number of measurements to confirm the findings from camera systems and now also magnetic contacts, which speak to the fact that these devices can work fully beyond the range of operating temperatures according to the manufacturer.

## Figures and Tables

**Table 1:** MAS 203 magnetic contact on and off values during experimental tests.

Temperature [°C]	Clamping Distance [mm]	Standard Deviation	Opening Distance [mm]	Standard Deviation
−40	28.5	1.96	28.5	1.74
−35	29	1.93	29	1.08
−30	29.5	0.15	29.5	0.23
−25	30	1.97	30	1.79
−20	31	0.35	31	1.62
−15	32	0.45	32	0.34
−10	33	1.41	33	1.62
−5	34	0.91	34	0.35
0	34	0.45	34	0.87
10	34.5	1.41	34.5	0.30
20	35	0.41	35	0.63
30	35	0.22	35	0.91
40	34	0.34	34	1.86
50	34	1.10	34	0.20
60	34	0.87	34	0.21
70	34	1.21	34	1.24

**Table 2:** MAS 333 magnetic contact on and off values during experimental tests.

Temperature (°C)	Clamping Distance (mm)	Standard Deviation	Opening Distance (mm)	Standard Deviation
−40	22	0.53	23.5	0.33
−35	22	0.43	24	0.30
−30	22.5	1.78	24	1.86
−25	23	0.84	25	0.79
−20	23	1.18	26	0.87
−15	23	1.15	26	0.42
−10	23	1.19	25	0.25
−5	23	0.34	25	0.58
0	23	0.29	25	1.86
10	23.5	1.83	25	0.75
20	23	0.47	25	1.69
30	22.5	1.59	25	1.97
40	22.5	1.20	24.5	1.95
50	22	1.92	24.5	0.66
60	22	1.49	24	0.43
70	22	1.69	24.5	0.46

**Table 3:** SA 220 magnetic contact on and off values during experimental tests.

Temperature (°C)	Clamping Distance (mm)	Standard Deviation	Opening Distance (mm)	Standard Deviation
−40	54	3.35	57	3.39
−35	54.5	2.78	57.5	4.16
−30	54.5	3.77	58.5	2.45
−25	56.5	2.53	59	4.94
−20	58	4.03	60.5	2.74
−15	59	3.45	62	4.83
−10	61	4.87	64	2.62
−5	63.5	3.16	66	3.07
0	66	4.50	69	2.91
10	72	3.94	76	4.94
20	81.5	3.90	86	4.91
30	84	4.69	89	3.01
40	85	2.03	90.5	3.95
50	87	3.34	92	3.50
60	88	4.54	94	3.27
70	89	2.70	94	2.02

**Table 4:** USP 131 magnetic contact on and off values during experimental tests.

Temperature (°C)	Clamping Distance (mm)	Standard Deviation	Opening Distance (mm)	Standard Deviation
−40	21	1.11	16	1.09
−35	21	1.13	16	0.55
−30	21	0.91	16	0.60
−25	21	1.75	16.5	0.63
−20	21	0.87	16.5	1.48
−15	21	1.92	16.5	1.40
−10	21	0.63	16.5	0.66
−5	21	1.45	16.5	2.00
0	21.5	0.39	16.5	1.34
10	22	1.91	17	0.47
20	21.5	1.16	17	1.45
30	22	1.85	17	1.84
40	22	1.94	16.5	1.96
50	21.5	1.46	16	1.97
60	22	1.36	16	0.99
70	22	0.32	16	1.19

**Table 5:** USP 500 magnetic contact on and off values during experimental tests.

Temperature [°C]	Clamping Distance [mm]	Standard Deviation	Opening Distance [mm]	Standard Deviation
−40	47.5	2.53	48.8	2.03
−35	48.5	2.37	49.5	2.69
−30	50.5	3.63	52	3.34
−25	51.5	3.26	52.5	2.03
−20	52.5	2.73	54	2.13
−15	53.5	2.73	55	3.75
−10	54.5	2.57	56	2.39
−5	55.5	2.54	56.5	2.79
0	56.5	3.76	58	3.72
10	55.5	2.08	57	2.15
20	56.5	3.12	58	2.20
30	57.5	2.52	59.5	3.45
40	58.5	2.03	60.5	2.67
50	58.5	2.55	60.5	3.49
60	58.5	2.17	60	2.92
70	57.5	3.72	59.5	2.21

**Table 6:** Measured values of magnetic field strength.

Temperature	−10	−5	0	15	25	35
Arithmetic mean of measured values [mm]	11.91	11.92	11.92	11.92	11.92	11.92
Standard deviation	0.09	0.07	0.08	0.07	0.08	0.07

## Data Availability

Data is contained within the article.
